# Clinical Warburg Effect in a Patient With Mantle Cell Lymphoma: A Case Report

**DOI:** 10.7759/cureus.58768

**Published:** 2024-04-22

**Authors:** Himanshu Patel, Awais Paracha, Adit Singhal, Kevin Wang, Nouneh Gostanian

**Affiliations:** 1 Internal Medicine, Northwell, New Hyde Park, USA; 2 Internal Medicine, Zucker School of Medicine at Hofstra/Northwell, Hempstead, USA; 3 Hematology and Oncology, Northwell, New Hyde Park, USA; 4 Hematology and Oncology, Northwell Health Cancer Institute, Lake Success, USA

**Keywords:** hemodialysis, mantle cell lymphoma, lactic acidosis, hypoglycemia, warburg effect

## Abstract

The clinical Warburg effect is a rare occurrence in cancer biology where tumor cells primarily utilize glycolysis for energy production, leading to significant hypoglycemia and lactate formation. This presentation is associated with a poor prognosis for the patient. In this context, we describe the case of a 53-year-old woman with stage IV mantle cell lymphoma who developed the clinical Warburg effect with solely arrhythmia and without neurological symptoms. She received prompt treatment for glucose stabilization and underwent inpatient chemotherapy. This case underscores the importance of early intervention to reduce tumor burden and highlights the effectiveness of hemodialysis in stabilizing metabolic acidosis. Further investigation into this approach is warranted.

## Introduction

The Warburg effect entails cancer cells relying heavily on glucose metabolism and glycolysis, even in aerobic conditions [1]. This phenomenon is attributed to the heightened demand for tumor cells to promote glycolysis intermediates for cell division and tumor expansion [1]. Consequently, lactic acidosis ensues as nicotinamide adenine dinucleotide, and hydrogen (NADH) is recycled to NAD+ to sustain adenosine triphosphate (ATP) production. 

The clinical Warburg effect (CWE) is a rare occurrence primarily observed in an already high-risk group of patients with advanced leukemia, lymphoma, or solid malignancies [2]. However, CWE itself imposes a more dire prognosis, with a 70% mortality rate within one year of diagnosis and a hazard ratio of 3.87 for one-year mortality compared to patients with advanced hematologic malignancies without CWE [3]. This underscores the importance of emergent oncologic evaluation and early intervention. In this context, we present a case involving a patient recently diagnosed with mantle cell lymphoma (MCL) who developed hypoglycemia and lactic acidosis. 

## Case presentation

A 53-year-old woman with a medical history including a repaired tetralogy of Fallot, hypertension, a significant smoking history of 30 pack-years, hormone-positive invasive ductal carcinoma of the right breast on anastrozole post lumpectomy, and adjuvant radiation therapy of the right breast, along with a diagnosis of MCL, presented with bilateral leg swelling and edematous pain. These symptoms had progressively worsened over the prior three months, leading to a decline from an active to a sedentary lifestyle. Consequently, she required assistance from her family to perform activities of daily living. 

Notably, she had been found to have MCL incidentally on an axillary lymph node dissection nine months before her presentation. The diagnosis of MCL was then confirmed by a bone marrow biopsy six months prior to presentation. However, she had not started treatment due to a lack of follow-up with her oncologist. 

**Figure 1:**
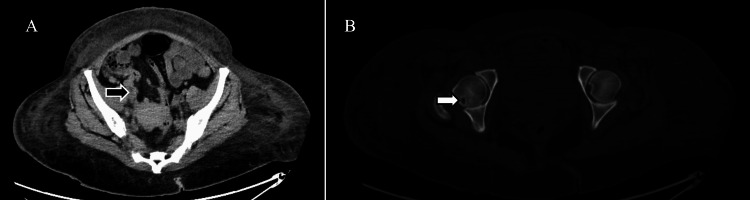
A CT scan shows evidence of metastatic mantle cell lymphoma. A: Representative image of lymphadenopathy (black arrow) measuring 1.6 cm; B: Lytic bone lesion (white arrow) measuring 0.8 cm.

On admission, her MCL International Prognostic Index (MIPI) score was 6.8 (age 53, Eastern Cooperative Oncology Group (ECOG) Performance Status 2, serum lactate dehydrogenase (LDH) of 383, white blood cell count of 14.63 K/uL, and Ki67 status of 5%), indicating poor prognosis. However, treatment for her MCL was not immediately initiated to allow for stabilization of her renal disease and further assessment of her MCL status given the significantly increased lymphadenopathy on a computed tomography (CT) scan (Figure 1A) on admission compared to those at the time of the initial diagnosis. As the workup of her MCL was conducted, she was treated for her lower extremity edema, initially with bumetanide 2 mg twice daily. Hemodialysis was initiated on hospital day three, and subsequently, a renal biopsy was performed on day 23 to determine the cause of her acute renal failure. She was confirmed to have indolent, stage IV, classic nodal MCL on day 25 of her hospitalization based on genetics, a Ki67 status of 5%, imaging (Figures 1A, 1B), and biopsy-proven involvement of the kidney as well as the bone marrow. 

On day 27 of her hospitalization, a rapid response was initiated due to supraventricular tachycardia (SVT) with a heart rate of 180 beats/min. During the incident, she was afebrile, normotensive, and exhibited an oxygen saturation of 100% on room air. However, the patient had multiple hypoglycemic readings (lows of 34 mg/dL on fingerstick blood glucose, 50 mg/dL on peripheral blood draw, and 31 mg/dL on venous blood gas), even though her blood glucose had been normal prior to this episode. During these readings, the patient had no explicit symptoms and was mentating normally. Additionally, a significant high anion gap metabolic acidosis (HAGMA) was noted, characterized by an anion gap of 24 mmol/L, a bicarbonate of 16 mmol/L, and a lactate of 16.6 mmol/L with no new transaminitis. Notably, there were no signs of sepsis, ischemia, hypoperfusion, hypoxemia, seizures, or bleeding, and CT imaging revealed no notable findings. Subsequent repeat electrocardiograms and cardiac biomarkers, conducted after rate control with metoprolol (5 mg), were not indicative of an ischemic process. Collectively, these findings suggested the presence of type B lactic acidosis, raising concerns for CWE given her history of stage IV MCL [3,4].

The hypoglycemia was treated with a bolus of 50 mL of 50% dextrose and an infusion of 10% dextrose at 50 mL/hr. She had subsequent episodes over the next week, for which she received oral glucose and infusions of dextrose as needed to maintain her blood sugar. To counteract her acidosis, she received 650 mg of sodium bicarbonate three times daily until her bicarbonate levels were corrected. Furthermore, she continued to undergo hemodialysis to address the acute renal failure identified during her initial presentation, which a biopsy showed to be due to interstitial fibrosis and tubular atrophy. 

**Figure 2:**
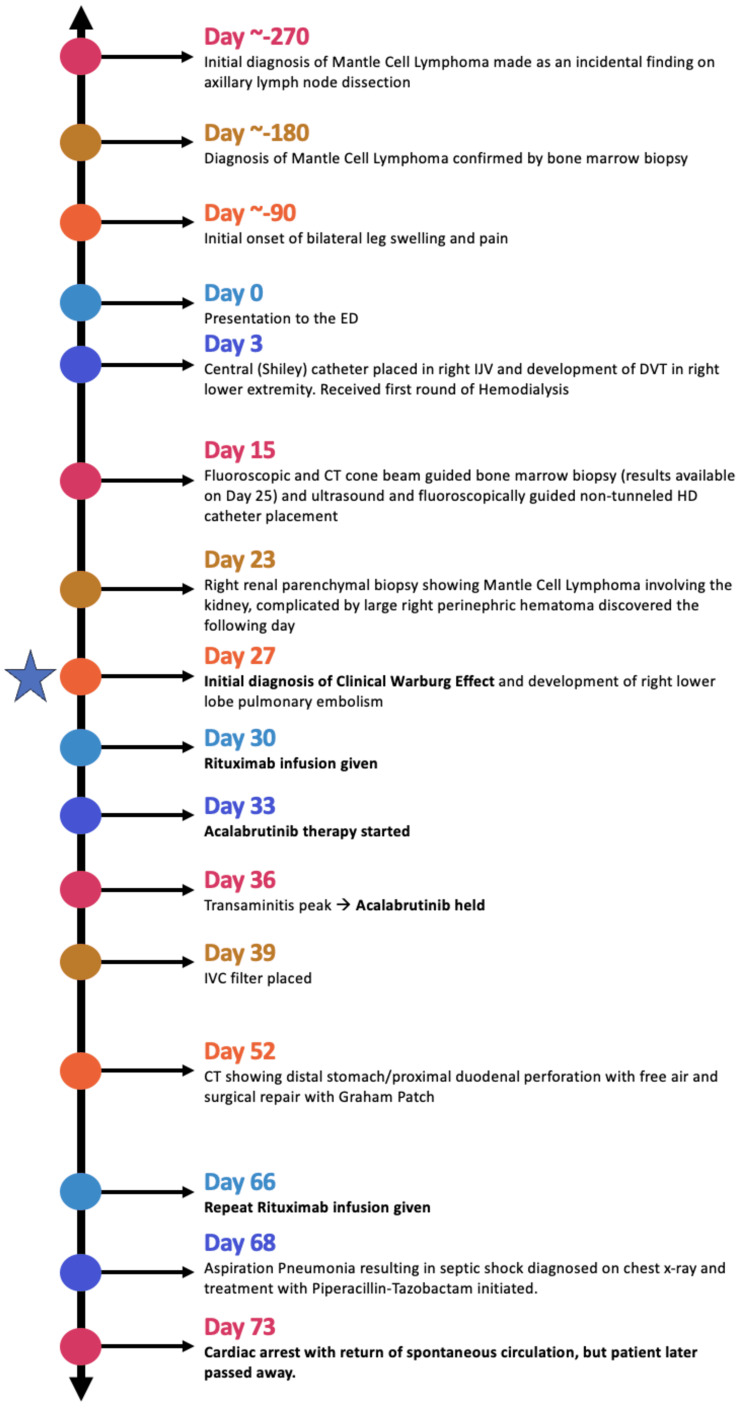
Timeline of events relevant to the patient's diagnosis and treatment of mantle cell lymphoma and CWE, as well as significant complications. CWE: clinical Warburg effect; DVT: deep vein thrombosis; HD: hemodialysis; IVC: inferior vena cava; IJV: internal jugular vein Image Credits: Himanshu Patel

**Figure 3:**
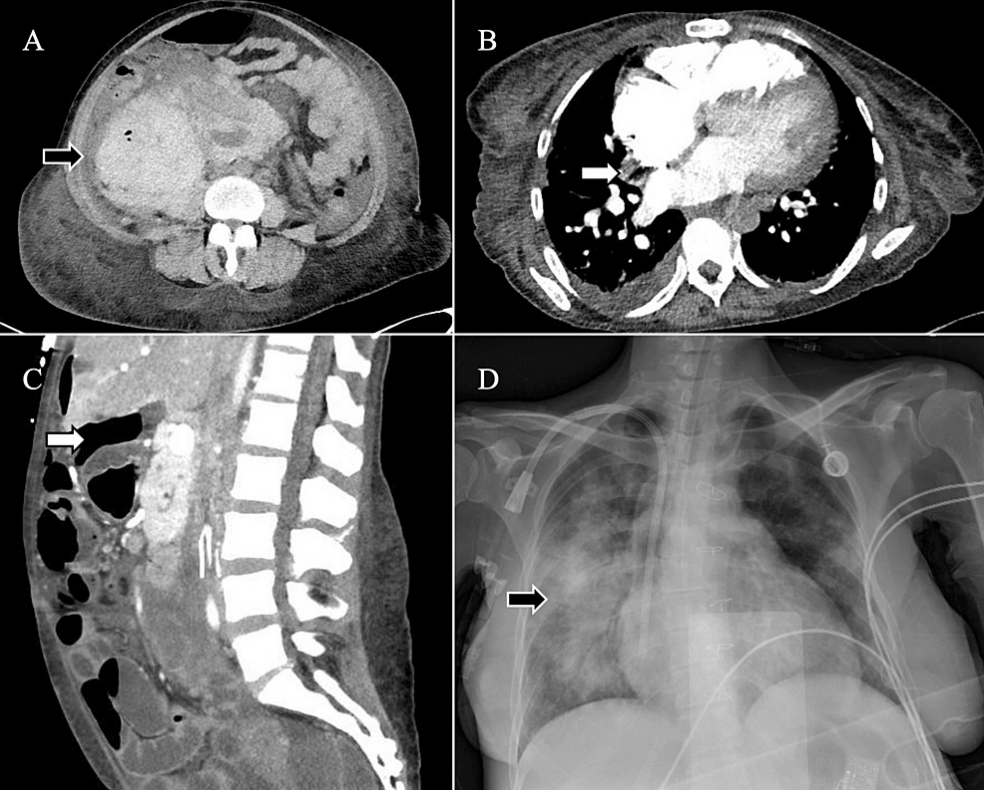
Imaging findings of complications during the patient's hospital course. A: Right perinephric hematoma (black arrow) measuring 15.8 x 10.7 cm; B: Filling defect within the subsegmental branch of the right lower lobe pulmonary artery (white arrow); C: Sagittal image showing pneumoperitoneum (white arrow); D: Chest radiography (CXR) showing right perihilar opacities (black arrow).

Given the extensive nature of her disease and the manifestation of CWE, inpatient chemotherapy for MCL was initiated. She received rituximab 375 mg/m2 twice five weeks apart (days 30 and 66) and acalabrutinib 100 mg twice daily, beginning on day 33. Considering acalabrutinib’s potential to decrease platelet production and her already low platelet count of 64 K/uL, the patient was discontinued from anticoagulation due to the risk of hemorrhage. Due to her history of deep vein thrombosis and pulmonary embolism (Figures 2, 3B), an inferior vena cava filter was inserted. Though she did not develop tumor lysis syndrome, allopurinol 100 mg three times per week was initiated based on her estimated glomerular filtration rate (eGFR) for prophylaxis. 

She began to develop progressively worsening transaminitis on hospital day 26, with an aspartate aminotransferase (AST) that peaked at 2088 U/L and an alanine transaminase (ALT) of 235 U/L on day 36 of her hospitalization. This transaminitis occurred in the context of significant bleeding during the patient’s renal biopsy, which was performed on day 23 of the patient’s hospitalization (Figure 2), after which a CT showed a large right perinephric hematoma (Figure 3A). Given a 2.1-point drop in her hemoglobin and a serum creatine kinase of 916 U/L, she likely developed shock liver and subsequent transaminitis. Her acalabrutinib was initially held starting on day 36 to prevent further worsening of her transaminitis, and it was never restarted due to the development of hyperbilirubinemia. 

During the hospital stay, follow-up imaging post-chemotherapy revealed a notable reduction (up to 30%) in the size of some lymph nodes. However, the patient encountered complications (Figure 2), including a perforated gastric ulcer (Figure 3C) that required surgical repair with a Graham patch, as well as aspiration pneumonia (Figure 3D). Subsequently, a cardiac arrest occurred, but the patient experienced a return of spontaneous circulation and was transferred to the medical intensive care unit (MICU). In the MICU, she underwent intubation and required maximal IV norepinephrine for blood pressure support. Despite these efforts, her condition continued to decline, leading to her passing. 

## Discussion

This case underscores a unique manifestation of CWE in a patient with both MCL and breast cancer, where the patient experienced hypoglycemia with a level as low as 31 mg/dL, with SVT being the sole symptom. 

Ordinarily, when blood glucose drops below 50 mg/dL, neuroglycopenic symptoms begin to emerge, ranging from agitation to death [5,6]. Despite the potential severity of hypoglycemia, there are reported instances of CWE in which patients reached levels as low as 9 mg/dL without exhibiting symptoms [7]. This phenomenon is attributed to a partially defined metabolic pathway in the brain, wherein lactate is converted to pyruvate and subsequently utilized for energy production [8]. This relies on a unique aspect of the CWE, in which tumors produce excess lactate that is available for the brain's energy requirements. 

While hypoglycemia is primarily associated with neurologic symptoms, it is also known to present with arrhythmia, which, while less common, is a major cause of sudden death in hypoglycemia [9]. Arrhythmias often present with an increased heart rate, which is believed to be due to increased activity of the sympathoadrenal system [10], which is further supported by the ability of adrenergic antagonists to mask the autonomic findings of hypoglycemia. 

Considering our patient's concurrent diagnoses of MCL and breast cancer, determining the exact cause of her CWE posed a challenge. Hematologic malignancies are commonly associated with CWE as compared to solid tumors, based on the current literature review [2]. Additionally, the substantial tumor burden of her MCL compared to her breast cancer, which had been treated with lumpectomy, adjuvant radiation therapy, and anastrozole suggests MCL was the more likely cause of her CWE. Finally, the patient’s episodic hypoglycemia resolved after the initiation of chemotherapy for her MCL. 

Previous case reports have underscored the importance of early treatment involving chemotherapy, glucose, and bicarbonate in addressing CWE [11,12]. The patient was promptly initiated on chemotherapy with rituximab and acalabrutinib after she presented with CWE, despite the initial plan to begin treatment after her renal failure and anemia due to blood loss from her renal biopsy improved. Rituximab and acalabrutinib were chosen instead of more conventional combinations such as rituximab and bendamustine to prevent exacerbation of the patient’s renal and hepatic impairments. 

Hemodialysis has also been suggested as a potential therapy that requires further exploration because bicarbonate alone, which treats acidosis, does not correct hyperlactatemia and potentially exacerbates it [7,13]. Hence, a combination of bicarbonate and hemodialysis was utilized to promptly and effectively rectify acidosis and hyperlactatemia while maintaining homeostatic blood pH between hemodialysis sessions. The initiation of hemodialysis in this patient even before the manifestation of CWE may have contributed to delaying further decline in her condition by reducing the extent of acidosis and hyperlactatemia she experienced. Consequently, in addition to being an oncologic emergency necessitating emergent evaluation and intervention to reduce tumor burden, we posit that the potential prompt initiation of hemodialysis in the context of CWE could enhance outcomes and merit further investigation. 

## Conclusions

We present a case involving the CWE observed in a patient diagnosed with stage IV MCL, highlighting a patient who presented with arrhythmia and, notably, did not have any neurologic symptoms. Considering the notable morbidity associated with this phenomenon, CWE should prompt an immediate oncologic assessment and intervention. We suggest that hemodialysis could serve as a supplementary treatment, potentially enhancing outcomes by not only addressing metabolic acidosis but also eliminating lactic acid from the body. Thus, further comprehensive research on this potential intervention is warranted. 
